# Immunological Insights into Photodynamic Therapy of Glioblastoma Multiforme

**DOI:** 10.3390/molecules30153091

**Published:** 2025-07-24

**Authors:** Paweł Woźnicki, Dorota Bartusik-Aebisher, Agnieszka Przygórzewska, David Aebisher

**Affiliations:** 1 Doctoral School, Medical College, University of Rzeszów, 35-310 Rzeszów, Poland; pawelw@dokt.ur.edu.pl; 2 Department of Biochemistry and General Chemistry, Medical College, University of Rzeszów, 35-310 Rzeszów, Poland; dbartusikaebisher@ur.edu.pl; 3 English Division Science Club, Medical College, University of Rzeszów, 35-310 Rzeszów, Poland; ap117623@stud.ur.edu.pl; 4 Department of Photomedicine and Physical Chemistry, Medical College, University of Rzeszów, 35-310 Rzeszów, Poland

**Keywords:** glioblastoma multiforme, photodynamic therapy, immunotherapy

## Abstract

The Gliomas account for 81% of all malignant central nervous system tumors and are classified by WHO into four grades of malignancy. Glioblastoma multiforme (GBM), the most common grade IV glioma, exhibits an extremely aggressive phenotype and a dismal five-year survival rate of only 6%, underscoring the urgent need for novel therapeutic approaches. Immunotherapy has emerged as a promising strategy, and photodynamic therapy (PDT) in particular has attracted attention for its dual cytotoxic and immunostimulatory effects. In GBM models, PDT induces immunogenic cell death characterized by the release of damage-associated molecular patterns (DAMPs), which promote antigen presentation and activate T cell responses. Additionally, PDT transiently increases blood–brain barrier permeability, facilitating immune cell infiltration into the tumor microenvironment, and enhances clearance of waste products via stimulation of meningeal lymphatic vessels. Importantly, PDT can reprogram or inactivate immunosuppressive tumor-associated macrophages, thereby counteracting the pro-tumoral microenvironment. Despite these encouraging findings, further preclinical and clinical studies are required to elucidate PDT’s underlying immunological mechanisms fully and to optimize treatment regimens that maximize its efficacy as part of integrated immunotherapeutic strategies against GBM.

## 1. Introduction

Gliomas are the most common primary brain tumors, accounting for 81% of malignant tumors of the central nervous system [1]. According to the World Health Organization classification, these tumors are divided into four grades, among which grade 1 and 2 gliomas denote low-grade malignancies, and grade 3 and 4 gliomas denote high-grade malignancies [2]. Typically, a relatively high grade is associated with a poor prognosis [3]. The most common grade 4 glioma is glioblastoma multiforme (GBM). The five-year survival rate for patients with this tumor is only 6% [4]. The current standard of therapy, which includes total resection, radiation therapy in the focal tumor area, and concurrent chemotherapy with temozolomide and a specific dose of radiation therapy [5,6], is therefore far from sufficient, and new forms of therapy are constantly being sought [7]. One of these is photodynamic therapy (PDT). PDT is based on the local or systemic application of a photosensitive compound, a photosensitizer (PS), which is intensively accumulated in pathological tissues. Photosensitizer molecules absorb light of the appropriate wavelength, initiating activation processes that lead to the selective destruction of abnormal cells [8]. The effect of PDT against GBM has been extensively studied over the past 30 years, in preclinical and clinical trials with promising results [9]. Recent years of work on PDT have shown that the therapy is also effective in activating the immune system [10], which has led it to be tested as a form of immunotherapy [11]. Immunotherapy, which aims to boost the host immune system to provide passive or active immunity against malignant tumors, has ushered in a new era in cancer treatment [12]. Despite many challenges, various forms of immunotherapy are emerging as promising avenues that may offer new hope for the treatment of GBM [13]. Thus, in light of these data, PDT’s ability to activate the immune system seems particularly attractive. In our work, we provide immunological insights into GBM PDT. We summarize the evidence for the ability of PDT to activate the immune system against GBM. We also present the effects of GBM PDT on blood–brain barrier (BBB) permeability, immunosuppress tumor-associated macrophages.

## 2. Photodynamic Therapy of Glioblastoma Multiforme Induces Immune System Activation

### 2.1. Photodynamic Therapy Induces Immunogenic GBM Cell Death and the Release of DAMP Signaling Molecules

The first step in the induction of an immune response by PDT is the induction of immunogenic cell death (ICD) and the release of tumor antigens along with molecules known as damage-associated molecular patterns (DAMPs) [14]. DAMPs are molecules that are normally confined to subcellular compartments of living cells, such as the nucleus, cytosol or biological membranes. In response to stress, damage or cell death, they become exposed and secreted, acquiring immunostimulatory properties [15]. This allows the maturation and activation of antigen-presenting cells, which phagocytose dying tumor cells and mediate the initiation of an immune response [16]. The ability of PDT to induce DAMP release from both human GBM cells and mouse models of GBM has been well documented. PSs for which this ability has been directly demonstrated in vitro include protoporphyrin IX (PpIX), a photoactive metabolite of 5-aminolevulinic acid (5-ALA) [17], a mixture of di-, tri- and tetrasubstituted fractions of aluminum phthalocyanine, when the number of sulfo groups is 3.4 (Photosens, PHS), bis-*N*-methylglucamine salt of chlorin e6 (Photoditazine, PD) [18], and tetra(aryl)porphyrin dyes with 9-phenanthrenyl (Pz-I) and 4-(4-fluorobenzyloxy)phenyl (Pz-III) groups in the aryl backbone of the macrocycle [19]. These photosensitizers showed diverse subcellular localization, with PpIX (produced from 5-ALA) accumulating mainly in mitochondria [20], Pz-I, Pz-III, and Photodithiazine localizing in endoplasmic reticulum and Golgi apparatus [18,19], and Photosens concentrating in lysosomes and intracellular vesicles [18]. This pattern of PS distribution indicates that ICD activation of GBM cells is independent of the subcellular localization of PS, which is consistent with previous findings [10]. However, it should be noted that the accumulation of photosensitizers under in vivo conditions may vary depending on their properties. Chlorin e6-based photosensitizers tend to preferentially accumulate in the endothelium of tumor blood vessels [21]. Increased transcription of DAMPs may already occur at the stage of photodynamic damage, before they are released from the cells. Kammerer et al. showed that 4 h after 5-ALA-PDT, the most strongly induced and expressed genes of GBM cell lines U87 and U373 were those encoding heat shock protein 70 (HSP-70). Their dominance was lost 24 h after 5-ALA-PDT [22]. DAMPs released by the aforementioned PSs included HSP-70 [17], calreticulin (CRT) [18], HMGB1, and ATP [18,19], as summarized in Table 1.

The dynamics of DAMP release vary depending on the amount of time after PDT. Turubanova et al. demonstrated CRT exposure even before the cell membrane of GL261 cells ruptured at 1.5–3 h after PHS-PDT or PD-PDT treatment [18]. CRT regulation on the surface of these cells was more pronounced than after treatment with mitoxantrone [18], which is a known inducer of ICD and serves as a positive control in studies [24,25]. HMGB1 and ATP were released with the rupture of the plasma membrane [18]. Since antigen-presenting cell activation is a key step in the induction of the adaptive immune response after PDT [14] and the primary mechanism of this process is the release of DAMP [26], it can be speculated that these PSs also exhibit the ability to release DAMP and tumor antigens. Such PSs include aluminum phthalocyanine disulfonate (AlPcS2a) [27,28], tetrahydroporphyrin-tetrathosylate (THTPS) [29], hematoporphyrin monomethyl ether (HMME) [30], and Pd-bacteriophosphoribidate [31]. Importantly, 5-ALA-PDT and talaporfin sodium-PDT (TPS-PDT) can also induce GBM cell necroptosis [32,33,34], a regulated form of necrosis that depends on phosphorylation of a mixed kinase-like kinase by receptor-interacting kinases-1 and 3 [35]. This lytic form of cell death optimizes antigen delivery and adjuvant to immune cells, potentially enhancing the efficacy of anticancer treatment through a combination of cell suicide and immune response [36].

### 2.2. Photodynamic Therapy of GBM Induces Non-Specific Immune System Response

Among the first cells recruited to areas illuminated during PDT are neutrophils. This process occurs as early as a few minutes after illumination, which is associated with the gradual constriction of endothelial cells and exposure of the subendothelial matrix that allows neutrophils to adhere to the walls of postcapillary veins [37]. The presence of neutrophils in therapeutic areas of GBM PDT has been confirmed in studies in mouse models [38,39,40]. Neutrophils have multiple functions: they are involved in the direct killing of tumor cells, activation of other immune cells, and are a source of numerous pro-inflammatory mediators [41]. A second population of cells that infiltrate tumors early after PDT are monocytes and macrophages [42]. The presence of macrophages in the therapeutic area of a GBM after PDT was demonstrated by Akimoto et al. in all of the patients they studied. In that study, PDT was used in combination with other therapeutic modalities, such as surgical resection, temozolomide chemotherapy, radiation therapy, bevacizumab or boron neutron capture therapy [43]. Macrophages not only infiltrate areas of damage but play a key role in activating the immune response, including antigen presentation [44]. As in other cancers [45], complement C3 has been identified as an important chemoattractant in the advanced phase of PDT-induced inflammatory infiltration [46]. It was noted that the median survival time of nude mice with G422 GBM deficient in complement C3 after PDT was only 18.6 ± 5.8 days, while immunocompetent mice survived an average of 44.3 ± 6.0 days [46]. In addition to neutrophils and macrophages, dendritic cells (DCs) are also involved in the nonspecific response [47] and are a key component of the transition between nonspecific and specific responses. Activation of the nonspecific immune response is essential for the subsequent induction of the adaptive arm of the immune system after PDT [48], as shown below.

### 2.3. DAMPs Released During Photodynamic Therapy of GBM Induce Activation of Antigen-Presenting Cells

As previously mentioned, DAMPs induce the activation and maturation of antigen-presenting cells [16], a key step in the generation of an immune response after PDT [14]. Etminan et al. indicate that HSP-70 plays a particularly important role in the processes of DC activation and maturation after PDT. Inhibition of HSP-70 on spheroids treated with 5-ALA-PDT prior to evaluation of tumor material uptake from spheroids significantly inhibited tumor antigen uptake and DC maturation, as indicated by a significant decrease in the frequency of CD83+ mature DCs [17,49]. Antigen-presenting cells are responsible for the capture and processing of antigens, the presentation of these processed antigens to T cells, and the delivery of costimulatory signals that stimulate their proliferation [50]. Among the antigen-presenting cells described so far are DCs [51], macrophages [52], and B cells [53]. Of these, DCs play the most significant role in antigen presentation [26]. The effect of GBM PDT on DCs has so far been studied in the context of developing vaccines based on these cells. The mechanisms by which PDT-treated GBM cells interact with DCs are diverse. GBM cell lines U251 and U87, subjected to 5-ALA PDT, have been shown to significantly stimulate the migration of human immature DCs in their direction [17]. In addition, both human GBM cells and mouse models of GBM induce uptake of tumor material by DCs after PDT treatment [17,18,29,54]. Importantly, this process occurs only against dying tumor cells [18]. The rate of phagocytosis depends on the ratio of DCs to dead GBM cells. Increasing this ratio from 1:1 to 1:5 in the GL261 model led to a proportional increase in their engulfment rate [18]. In this work, cells were incubated with photosensitizer in serum-free medium [18], which could significantly increase cellular uptake and cytotoxicity, especially compared to conditions containing 10% fetal bovine serum—as has been shown for Sn(IV) chlorin e6, where serum reduced uptake by up to 60–75% [23]. PDT-treated human GBM cells and murine GBM models induce DC maturation [17,18,29,30], as evidenced by changes in the expression of surface markers such as CDD83+ [49], CD40+ [55], CD 80+, CD86+ [56], and OX-6 (MHC-II) [57]. This process requires the presence of dying GBM cells after PDT [18]. Turubanova et al. showed that the expression of the aforementioned markers was similar to that induced by lipopolysaccharide used as a positive control [18]. PDT-treated GBM cells induce increased functional DC activity [17]. Pulsing DCs with PDT/ionizing radiation-treated lysate significantly increased IL6 and TNFα release compared to ionizing radiation-treated samples after 24 h, indicating the ability of PDT to increase IL-6 and TNFa release in DCs [29]. DCs cultured with GL261 cells subjected to Photosens-PDT altered the expression program of genes related to cellular processes, biological regulation, metabolic processes, response to stimuli, signaling, developmental processes, multicellular organism processes, and immune system processes. In particular, high expression levels of Tgfb3, IL-6, IL-23, and Th17 lymphocyte activation genes were observed, while there were no significant changes in the expression of Th1, cytotoxic T lymphocytes and regulatory T cells cell markers [54].

Macrophages can also act as antigen-presenting cells after PDT of GBM cells. F98 cells subjected to AlPcS2a-PDT coincubated with 8383 macrophages induced changes in their morphology, leading to their enlargement and irregular shape, as well as an increase in the number of intracellular inclusions [28]. Macrophage vaccine administered to mice with GBM resulted in the generation of an immune response and a significant reduction in tumor size [27] or complete absence of tumor growth [28], indicating effective antigen presentation by macrophages. The number of CD68+ cells, including macrophages [58], increased 24 h after Hematoporphyrin Derivatives-PDT (HpD-PDT) and peaked 48 h after HpD-PDT in a mouse model in which PDT inhibited GBM growth through an anti-tumor response [46]. However, it should be noted that CD68+ expression is also characterized by Tumor-associated macrophages (TAM), which may function as an immune suppressor and contribute to GBM progression in the tumor microenvironment resulting in a worsened prognosis [59,60]. On the other hand, Saito et al. observed no significant increase in CD68+ cells after TPS-PDT [61]. This is perhaps due to differences in the therapeutic regimens used. This thesis is supported by a study by Hirshberg et al. in which the number of infiltrating macrophages in a mouse model of GBM increased with the use of lower fluence values (10 mW and 50 mW), indicating that therapeutic parameters can be manipulated to potentially increase the efficacy of therapy [38].

### 2.4. The Specific Immune Response Against GBM Induced by Photodynamic Therapy Occurs with T Cells

After maturation, antigen-presenting cells migrate to secondary lymphoid tissues, such as lymph nodes [62] or the spleen [63] where they interact with T [64,65] and B [66] lymphocytes. This is followed by a complex process that is the presentation of MHC/antigen complexes along with costimulatory molecules and the secretion of pro-inflammatory cytokines, which induces the corresponding immune response [67]. GBM PDT has shown the ability to activate T cells. In mouse models, HpD-PDT led to a significant increase in the levels of TNF-α and IFN-γ released by T cells from the spleen [46], providing evidence of their activation [68,69]. Moreover, HpD-PDT restored the reduced CD4+/CD8+ lymphocyte ratio to normal 72 h after treatment [46], suggesting activation of the systemic immune response [70]. Presumably, DCs also activate Th17 lymphocytes, as indicated by high expression levels of Th17 cell-activating genes in these cells, as described previously [54]. A key step in the generation of a response is the proliferation of T cells, both CD8+ and CD4+ in response to antigens [71]. Proliferation of these cells was observed during PDT of GBM. The lymph nodes of mice that were inoculated with DCs coincubated with PHS-PDT-treated GL261 cells contained significantly increased numbers of CD8+ T cells [54]. Stimulation of mouse CD4+ and CD8+ T cells with tumor lysate of GL261 cells or DCs pulsed with this lysate subjected to PDT induced T cell proliferation, whether applied before or after freeze/thaw cycles. It should be noted, however, that in this study, GBM cells were also treated with radiation therapy at a dose of 15 Gy [29].

The next step is the immune system response against the tumor [72,73]. The T lymphocyte-mediated immune response is the main response induced by PDT in the context of anti-tumor immunity [74]. In an autopsy study, all three GBM patients who received TPS-PDT in combination with other therapies (surgical resection, temozolomide chemotherapy, radiation therapy, bevacizumab, neutron therapy) showed the presence of T lymphocytes in the therapeutic area [43]. In contrast, a study by Saito et al. observed no significant differences in CD4+ and CD8+ cell counts in a mouse model of GBM despite the observed C6 cells death [61], although PDT with talaporfin sodium showed the ability to induce immunogenic cell death [75]. The ability of PDT to activate T cells against GBM has been well documented and is crucial to the anti-tumor effects of this therapy. In studies in immunodeficient T mice, median survival was shorter (24.8 ± 5.2 days) compared to immunocompetent BALB/c mice (44.3 ± 6.0 days) after PDT [46]. The ability of T lymphocytes after PDT to induce an immune response against GBM has also been demonstrated when administered to mice. Splenic T lymphocytes taken from nude mice that had been injected with GBM treated with PDT with intracerebral and subcutaneous G422 GBM significantly increased suppression of intracerebral and subcutaneous tumor growth [46]. Finally, this effect was also proven in vitro. Splenic T cells collected from PDT-treated GBM mice led to greater G422 and GL261 cells death in vitro than those collected from tumor-free or untreated PDT mice [46]. Th17 lymphocytes are also likely to be involved in the immune system response against GBM after PDT. After immunization with a vaccine prepared from DCs obtained by coincubation with PHS-PDT-treated GL261 cells, infiltration of IL-17+ cells was observed in the brains of mice. These results, combined with data on Th17 gene activation in DCs, suggest an important role for Th17 cells in activating the immune response [54]. In addition, inhibition of the orphan receptor-γt (RORγt), a regulator of the Th17 response and related to the retinoic acid receptor [76], significantly reduced the efficacy of DC vaccines and shortened the survival of mice, transforming the tumor microenvironment by reducing IL-17 levels in the tumor [54]. At the same time, PDT does not seem to have an adverse effect on the population of regulatory T cells. The percentage of CD152+ regulatory T cells in the T cell population after priming with pulsed DCs was minimal after the first priming (5.24 ± 0.02%) and was not increased compared to naive DCs (6.08 ± 0.04%). Moreover, the second priming of T cells did not significantly increase this percentage [29].

## 3. Photodynamic Therapy Opens the Blood–Brain Barrier and Induces Clearance of the Brain by Meningeal Lymphatic Vessels

**Figure 1:**
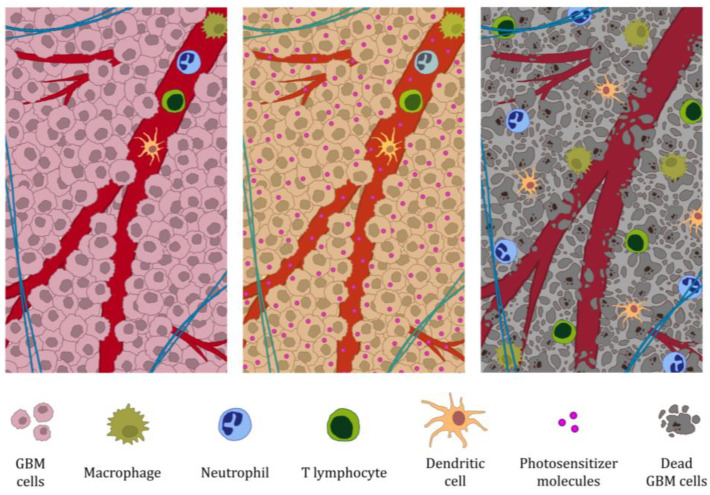
Opening the blood–brain barrier by photodynamic therapy for GBM. (1) All GBMs show clinically significant areas of tumor where the BBB remains intact [80]; (2) accumulation of photosensitizer in the tumor and its exposure;(3) opening of the BBB and increased infiltration of immune cells. The presence of macrophages and T lymphocytes in the therapeutic area of the GBM after PDT was demonstrated by Akimoto et al. in all patients studied. In this study, PDT was used in combination with other therapeutic modalities [43]. Presence of neutrophils in therapeutic PDT areas of GBM has been confirmed in studies in mouse models [38,39,40]. The presence of dendritic cells in the therapeutic area of GBM after PDT has not yet been confirmed. However, given PDT’s ability to induce a specific immune system response, including activation of T lymphocytes, it can be speculated that the therapy also increases blood–brain barrier BBB permeability for dendritic cells.

The blood–brain barrier is a complex vascular structure that separates the central nervous system from the peripheral blood circulation. It plays a key role in protecting brain tissue from blood-borne agents and is an important barrier to the passage of drugs and other exogenous compounds into the central nervous system [77]. However, the BBB is not completely airtight and allows for the entry of T lymphocytes and immune surveillance in the brain, challenging the earlier belief that this barrier is “hermetically sealed” [78]. Brain tumors disrupt the integrity of the BBB [79], but all GBM show clinically significant areas of tumor where the BBB remains intact [80]. In pathological situations, such as GBM and other cancers, the barrier function of the BBB is compromised, reducing its ability to limit the penetration of immune cells [81]. The ability of PDT to open the blood–brain barrier is well documented [82,83,84,85]. As shown previously after PDT of GBM, immune cells are observed in the therapeutic area. These cells, including macrophages, neutrophils, and lymphocytes are also observed in the PDT therapeutic area of the normal, intact brain, indicating the ability of PDT to increase the permeability of the BBB to these cells [84,86,87,88], potentially enhancing the immune system response against the tumor [46]. Moreover, by opening the BBB, PDT induces activation of the brain clearance process via the meningeal lymphatic system [89]. These structures are able to carry both fluid and immune cells from the cerebrospinal fluid and are connected to the deep cervical lymph nodes [90,91]. Meningeal lymphatic vessels play a key role in generating an effective immune response against brain tumors, and their ablation inhibits the immune response against these tumors [92]. The meningeal lymphatic system tocervical lymph node network contributes to the efficacy of radiotherapy in brain tumors and mediates radiotherapy-modulated anti-tumor immunity [93]. Thus, it can be speculated that by activating the clearance of the brain by the meningeal lymphatic system, PDT may enhance the host immune response against the tumor. On the other hand, Blokhina et al. showed that 5-ALA-PDT-induced extravasation of photo-induced 5-ALA from leaking blood vessels into the meninges causes photodamage to the meningeal lymphatic vessels, leading to a dramatic reduction in the network of these vessels and brain drainage [94]. The successive stages of BBB opening by PDT in GBM, from photosensitizer accumulation to barrier disruption to increased immune cell infiltration, are illustrated in Figure 1.

## 4. Photodynamic Therapy of GBM Impairs Tumor-Associated Macrophage Function

Tumor-associated macrophages are important components of the tumor microenvironment that promote processes involved in tumor progression [95]. Through a variety of mechanisms, TAMs also contribute to the development of GBM [96,97], and their presence is associated with a poor prognosis [98]. For this reason, they represent a promising therapeutic target for the treatment of these cancers [99]. TAMs also play an important role in the anticancer effects of PDT. These cells exhibit the highest intracellular levels of photosensitizers, which allows their selective destruction by PDT [100] and is likely due to the higher endocytosis capacity of M2 macrophages compared to M1 [101].

**Figure 2:**
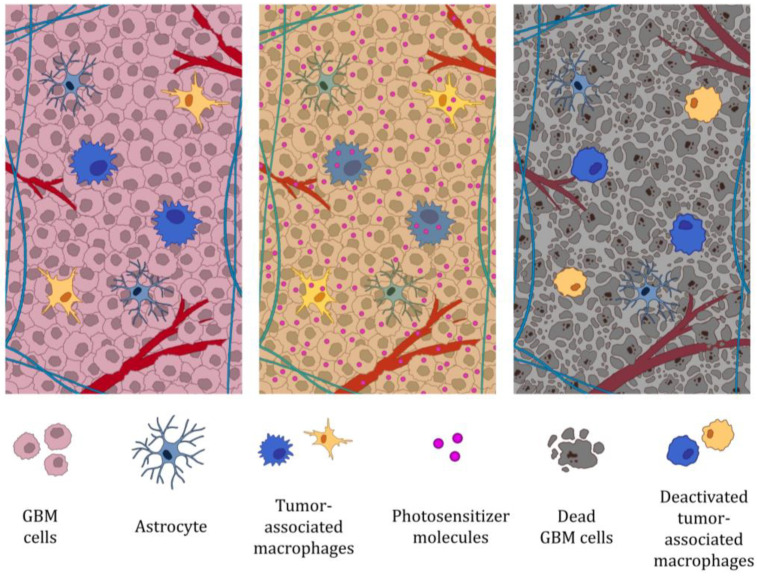
Photodynamic therapy of GBM impairs the function of tumor-associated macrophages; (1) Both microglia (resident CNS macrophages) and macrophages of peripheral origin constitute a population of TAMs and promote tumor progression [96,97]; (2) photosensitizer accumulation in the tumor. TAMs show the highest intracellular levels of photosensitizers [100]. Irradiation of the tumor; (3) TAM inactivation of the GBM [102].

The ability of PDT to inactivate TAMs has also been documented in GBM studies in vitro and in mouse models. Maklygina et al. showed that the application of 5-ALA-PDT leads to the accumulation of protoporphyrin IX in TAMs, allowing their precise inactivation [102]. In contrast, Lerouge et al. demonstrated that AGuIX@PS@KDKPPR nanoparticles targeting neuropilin-1 are internalized by macrophages with an M2 phenotype with higher efficiency than by M1 macrophages. PDT performed with these carriers resulted in selective death of M2 macrophages. Moreover, the application of AGuIX@PS@KDKPPR-PDT modifies the cell secretion profile of the U87 lineage towards macrophage polarization towards the M1 phenotype and, in an in vivo model, leads to an enhanced inflammatory response, disruption of blood–brain barrier integrity, and increased macrophage recruitment to the tumor [103]. The studies described confirm both the ability of PDT to inactivate TAMs and to selectively eliminate their M2 phenotype subpopulation in the GBM microenvironment, which may support an anti-tumor response. However, it should be noted that with regard to the actual anti-tumor response of PDT, macrophages can be considered a double-edged sword, as they can play a role in events critical for tumor destruction but also in events that promote tumor recurrence [100]. The mechanism by which PDT disrupts the activity of tumor-associated macrophages in GBM and leads to their inactivation is illustrated in Figure 2.

## 5. Conclusions

Despite intensive work to improve the efficacy of photodynamic therapy in the treatment of glioblastoma multiforme, the ability of this method to induce a strong and sustained immune system response remains under-researched and only partially understood. Available data are fragmentary, and many of the key mechanisms involved in the immune effects of PDT still need to be clarified in both experimental and clinical studies. It should be noted that most of the studies conducted to date have been preclinical, conducted mainly in vitro using GBM cell lines or in vivo on mouse models. While these models provide invaluable mechanistic insights, the complex nature of the human immune system and tumor microenvironment means that results obtained under such controlled conditions may not fully translate to clinical efficacy in patients. Moreover, some of PDT’s therapeutic effects—such as blood vessel closure and initiation of apoptosis—are often overlooked in immunological analyses. Meanwhile, it has been shown that certain forms of apoptosis, although traditionally considered immunologically “silent,” can, under certain conditions, lead to the induction of an immune response through the emission of DAMP signals such as calreticulin, ATP, and HMGB1 [104]. This gap underscores the need for cautious interpretation of preclinical results and highlights the urgent need for more comprehensive clinical trials to validate and optimize PDT-based immunotherapeutic approaches in GBM. Nevertheless, abundant preclinical and clinical evidence suggests that PDT of GBM can effectively initiate an immune response, particularly through the induction of immunogenic cell death, which facilitates the presentation of tumor-associated antigens to the immune system and the recruitment of antigen-presenting cells such as dendritic cells. In addition, the therapy can enhance immune effects by transiently increasing the permeability of the BBB, which allows better infiltration of immune cells into the tumor site and improves delivery of systemic agents, including immunotherapeutic drugs. Importantly, PDT can also promote mechanisms that counteract immunosuppression in the tumor microenvironment by modulating the phenotype of TAMs and reducing the population of regulatory T cells, thereby increasing the effector function of cytotoxic T lymphocytes.

Despite these promising immune effects, full success in generating a consistent and effective anti-tumor immune response has still not been achieved, limiting the ability of PDT to completely cure GBM. One of the key unresolved issues remains the variability of immune outcomes depending on the type of photosensitizer used, light parameters, treatment schedule and tumor heterogeneity. The key question remains: what strategies should be employed to effectively enhance the immune response in the context of GBM PDT? The answer to this question is not simple, if at all possible. To achieve this goal, it is necessary to use photosensitizers that induce not only effective destruction of tumor cells, but also potent and immunogenic cell death pathways. These should include the release of DAMPs and activation of innate immune pathways. Equally important is the precise design of therapeutic regimens that can multidimensionally affect the tumor microenvironment, overcoming local immune tolerance and promoting systemic anti-tumor immunity. Combining PDT with other therapeutic modalities, such as radiation therapy, immunotherapy with immune checkpoint inhibitors, anticancer vaccines or adoptive cellular therapies can further enhance the immunostimulatory effect and help overcome existing barriers to therapeutic success. In addition, the rapid development of nanotechnology [105] is opening up new perspectives; precisely designed nanoparticles can effectively deliver photosensitizers and immune adjuvants, enabling spatial–temporal control of drug release, better tumor selectivity, and improved modulation of the immune response.

Further research in this area is needed to fully realize the potential of PDT as an immunomodulatory therapeutic strategy for the treatment of GBM. Understanding the mechanisms underlying the PDT-induced immune response, identifying biomarkers of the response, and developing new synergistic therapeutic approaches could significantly improve outcomes for patients with this highly aggressive and refractory cancer.

## Figures and Tables

**Table 1:** Photosensitizers with directly demonstrated ability to induce DAMP release during photodynamic therapy of GBM cells.

DAMP	Photosensitizer	Photosensitizer Concentration [μM]	Incubation Time [h]	Fluence Value [J/cm^2^]	Cell Line	Reference
HSP-70	PpIX ^1^	95.3	4	No data available ^2^	U251	[17]
					U87	
CRT	PHS	1.4	4	20 J/cm^2^	GL261	[18] ^3^
	PD	1.2	4	20 J/cm^2^	GL261	[18]
HMGB1	PHS	1.4	4	20 J/cm^2^	GL261	[18]
	PD	1.2	4	20 J/cm^2^	GL261	[18]
	PZ-I	2.8	4	20 J/cm^2^	GL261	[19] ^4^
	PZ-III	1.7	4	20 J/cm^2^	GL261	[19]
ATP	PHS	1.4	4	20 J/cm^2^	GL261	[18]
	PD	1.2	4	20 J/cm^2^	GL261	[18]
	Pz-I	2.8	4	20 J/cm^2^	GL261	[19]
	Pz-III	1.7	4	20 J/cm^2^	GL261	[19]

^1^ In this study, PpIX was produced in cells by administration of 5-ALA; ^2^ the text by Etminan et al. does not specify the value of fluence used. However, it was reported that exposure of spheroids to laser light was performed for 625 s with 1 W which corresponds to a total delivered energy of 625 J [17]; ^3,4^ in the works cited ([18,19]), cells were incubated with photosensitizer in serum-free medium, which can significantly increase cellular uptake of photosensitizer [23].
